# Moderate Changes in CO_2_ Modulate the Firing of Neurons in the VTA and Substantia Nigra

**DOI:** 10.1016/j.isci.2020.101343

**Published:** 2020-07-04

**Authors:** Emily Hill, Nicholas Dale, Mark J. Wall

**Affiliations:** 1 School of Life Sciences, University of Warwick, Gibbet Hill, Coventry CV4 7AL, UK

**Keywords:** Molecular Neuroscience, Cellular Neuroscience

## Abstract

The substantia nigra (SN) and ventral tegmental area (VTA) are vital for the control of movement, goal-directed behavior, and encoding reward. Here we show that the firing of specific neuronal subtypes in these nuclei can be modulated by physiological changes in the partial pressure of carbon dioxide (PCO_2_). The resting conductance of substantia nigra dopaminergic neurons in young animals (postnatal days 7–10) and GABAergic neurons in the VTA is modulated by changes in the level of CO_2_. We provide several lines of evidence that this CO_2_-sensitive conductance results from connexin 26 (Cx26) hemichannel expression. Since the levels of PCO_2_ in the blood will vary depending on physiological activity and pathology, this suggests that changes in PCO_2_ could potentially modulate motor activity, reward behavior, and wakefulness.

## Highlights

• The substantia nigra (SN) and VTA are key to movement and goal-directed behavior
• Activity in specific SN and VTA neurons is modulated by physiological changes in CO_2_
• The neuronal CO_2_ sensitivity results from connexin 26 (Cx26) hemichannel expression
• Minute-to-minute changes in CO_2_ could modify motor activity and reward behavior

## Graphical Abstract

**Figure:**
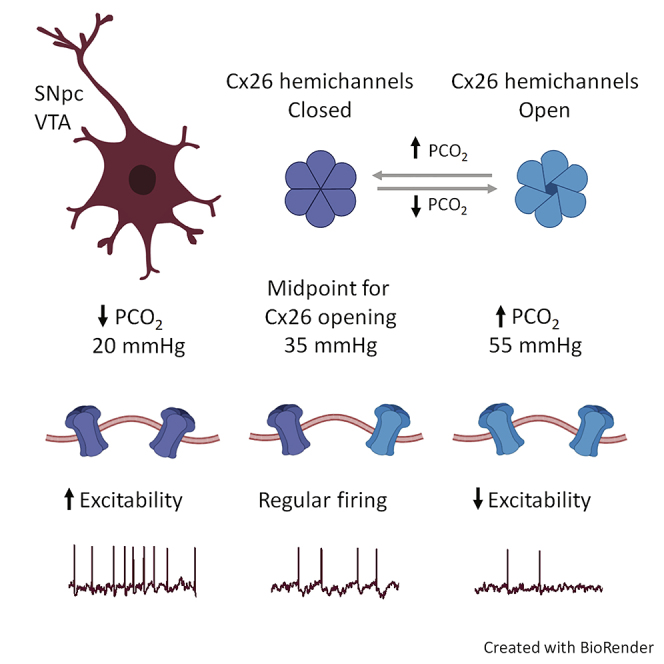

## Introduction

Carbon dioxide (CO_2_) is a waste product of cellular metabolism with its concentration in blood a major regulator of breathing. In humans, PCO_2_ in blood is normally ∼40 mm Hg but can be increased in conditions such as chronic obstructive pulmonary disease (COPD) and sleep apnea and can be decreased by hyperventilation and prolonged physical exertion. According to traditional consensus, CO_2_ is detected via the consequent change in pH, and pH is a sufficient stimulus for all adaptive changes in breathing in response to hypercapnia (Loeschcke, 1982). pH-sensitive ion channels and receptors have been proposed to play a role in respiratory chemosensing in both the periphery (carotid body) and centrally in the medullary chemosensory areas such as the retrotrapezoid nucleus and the medullary raphe (Trapp et al., 2008; Kumar et al., 2015; Wang et al., 2013; Hosford et al., 2018). pH sensing via ventral medullary glial cells may also contribute to the CO_2_-dependent regulation of breathing (Gourine et al., 2010; Turovsky et al., 2016). However, there is considerable evidence that CO_2_ can have additional independent effects from pH on central respiratory chemosensors (Eldridge et al., 1985; Shams, 1985). CO_2_ directly binds to connexin 26 (Cx26) via a structural motif, which results in carbamylation of Lys125, thus increasing hemichannel opening probability (Huckstepp et al., 2010a; Meigh et al., 2013). The midpoint for the binding is ∼40 mm Hg, which, as indicated above, is the resting level in human blood, and thus small changes in PCO_2_ will shift the open probability of Cx26 hemichannels. Pharmacological evidence suggests that Cx26 contributes to the CO_2_-dependent regulation of breathing (Gourine et al., 2005; Huckstepp et al., 2010b; Wenker et al., 2012), and this has recently gained support from genetic evidence that links binding of CO_2_ to Cx26 to the adaptive change in breathing (van de Wiel et al., 2020).

Coupling between dopaminergic neurons (DNs) in the substantia nigra (SN) was first described by Grace and Bunney (1983) who showed that the injection of lucifer yellow dye into single cells could result in the filling of neighboring “coupled” cells, with the dye transferring through gap junctions. They confirmed this using electrophysiology. Vandecasteele (2005) validated that pairs of DNs in the SNpc are coupled by functional gap junctions and later went on to describe the connexin expression profile of SN DNs (Vandecasteele et al., 2006). They reported that, in young rodents (postnatal day 7–10), these neurons express mRNA for Cx26 and Cx30, which are sensitive to CO_2_, but by P17–21 they only express mRNA for CO_2_-insensitive connexins (Vandecasteele et al., 2006). This observation led us to investigate whether the DNs in the SN of young rodents (P7–10) express CO_2_-sensitive hemichannels and thus have a CO_2_ phenotype. We subsequently discovered an additional population of neurons, GABAergic, in the ventral tegmental area (VTA), which also appear to express Cx26 hemichannels and are sensitive to CO_2_. Unlike the SN DNs, these neurons appear to retain their sensitivity to CO_2_ throughout development. Our findings reveal an unexpected role for CO_2_ in regulating the activity of these key brain regions and demonstrate a mechanism by which autonomic state could alter complex movement-related and goal-directed behaviors. This would also be the first documentation of connexin 26 hemichannel expression in neurons.

## Results

**Figure 1:**
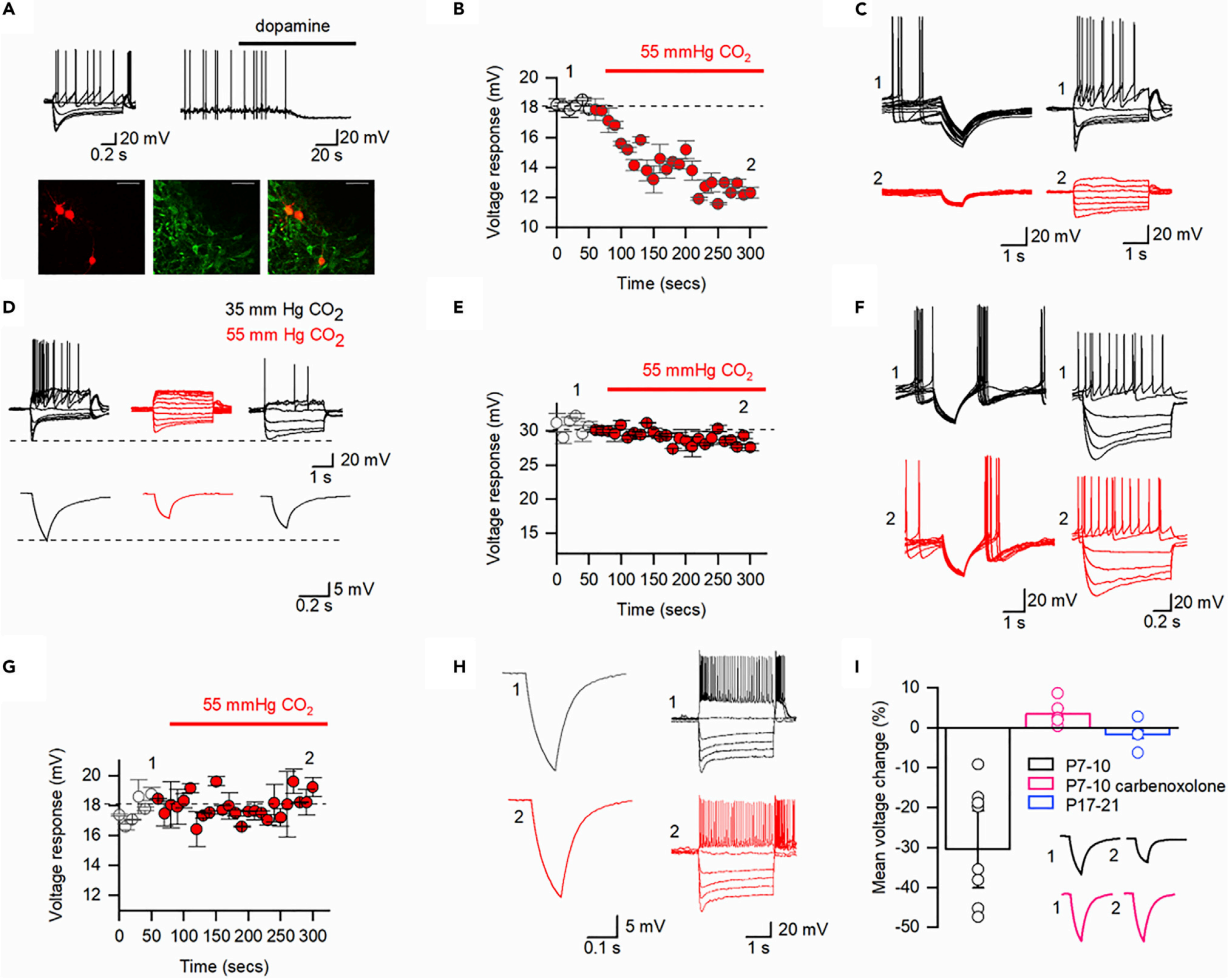
CO_2_ Sensitivity of Dopaminergic Neurons in the Substantia Nigra (A) Characteristic properties of SN DNs: voltage response to current injection, hyperpolarization in response to dopamine (30 μM) and recorded neurons (red) are TH^+^ (green). Scale bar, 100 μM. (B) Time course of changes in voltage response (P7–10, each point is a mean of six current steps, error bars are SEM) when CO_2_ was changed from 35 to 55 mm Hg. (C) Associated voltage traces (40 superimposed traces) and voltage responses to step currents at indicated time points from (B). (D) Representative voltage responses (P7–10) demonstrating input resistance and firing rate changes can be partially reversed (bottom traces are an average of 25 sweeps). (E) In P17–21 DNs there was no significant change in voltage response (each point is a mean of six current steps, error bars are SEM) when CO_2_ was changed from 35 to 55 mm Hg. (F) Associated voltage traces (40 superimposed traces) and voltage responses to step currents at indicated time points from (E). (G) If slices are incubated in carbenoxolone, there is no significant change in voltage response (P7–10, each point is mean of six current steps, error bars are SEM) when CO_2_ was changed from 35 to 55 mm Hg. (H) Associated mean voltage traces (average of 40 sweeps) and voltage responses to step currents at indicated time points from (G). (I) Quantification of voltage response changes (35–55 mm Hg CO_2_). Inset, control responses and responses in carbenoxolone (1, 35 mm Hg and 2, 55 mm Hg).

To investigate whether dopaminergic neurons (DNs) in the SN from P7–10 mice are sensitive to levels of carbon dioxide (CO_2_), as predicted from their connexin mRNA profile (Vandecasteele et al., 2006), we made whole-cell patch clamp recordings from DNs in acutely isolated slices. Putative DNs in the SN were identified by their electrophysiological profile. DNs were identified primarily by their position in the slice and characteristic current-voltage relationship; most displayed a large sag in response to hyperpolarizing current steps (characteristic of Ih), rebound and tonic firing at rest, and a hyperpolarizing response to dopamine application (Grace and Onn, 1989; Neuhoff et al., 2002). A subset of recorded neurons were confirmed as dopaminergic when positive for the dopamine marker tyrosine hydroxylase using immunohistochemistry (Grace and Onn, 1989; Figure 1A). In order to test whether the DNs were sensitive to CO_2_, following their identification with standard step current injections and in some neurons also the injection of naturalistic current (to measure firing rates), the level of CO_2_ (35 mm Hg, basal level) was increased to 55 mm Hg under isohydric conditions (compensatory changes in bicarbonate concentration to maintain constant extracellular pH during the CO_2_ stimulus, see Methods). This increase in PCO_2_ from 35 to 55 mm Hg (hypercapnia) produced a time-dependent reduction in the tonic firing rate and a reduction in the voltage change in response to hyperpolarizing current steps (Figures 1B and 1C). Both of these effects are characteristic of an increase in resting conductance. At steady state, the response to the hyperpolarizing current steps had fallen to 70 ± 9.6% of control (p = 0.0015, Figures 1B and 1C), the input resistance had fallen from 380 ± 28.15 to 217 ± 27.9 MΩ (p = 0.0027, *n* = 10), and the tonic firing was abolished. For a subset of recordings, we tested whether it was possible to get recovery when PCO_2_ was returned from 55 to 35 mm Hg; this was not quantified, but an example showing partial recovery of firing rate and input resistance is illustrated in Figure 1D.

These observations were not an artifact of the dialysis of the cell following whole-cell breakthrough as the cells were first allowed time to equilibrate, then standard and naturalistic currents were injected to form IV curves and to measure firing rates. In a subset of neurons, pharmacological agents such as dopamine were applied to identify the cells (∼30 min to apply and wash) prior to the alteration of CO_2_ and similar effects of changing the CO_2_ were observed. For initial controls, the experiment was first repeated without changing the PCO_2_ (although the solutions were still exchanged to eliminate any artifacts due to the mechanical process of solution change), and under these conditions, the resting conductance and firing rate of the neurons did not significantly change over the time course of the experiment (Figure S1). Second, the experiment was repeated with hippocampal CA1 pyramidal neurons and there was no significant change in the electrophysiological properties of these neurons with hypercapnia (voltage response was 100.3 ± 1.64% of control, p = 0.68, Figure S2, *n* = 6).

### Evidence That the Effects of PCO_2_ on Cell Conductance Are due to Cx26 Hemichannel Expression

We then took a number of approaches to investigate whether the SN DN CO_2_ sensitivity is the result of Cx26 hemichannel expression. First, as DNs in the SN of older mice (P17–21) do not express mRNA for CO_2_-sensitive connexins (Vandecasteele et al., 2006), they should therefore be insensitive to CO_2_ if it is connexin hemichannel dependent. Whole-cell recordings from DN in SN from P17–21 mice showed the expected changes in electrophysiological properties that have been reported (Dufour et al., 2014) to occur during postnatal development (Figure S3) but showed no significant response to increased PCO_2_ (voltage response was 101 ± 0.9% of control, p = 0.33, *n* = 4, Figures 1E and 1F). Second, the effects of increasing PCO_2_ could be blocked by the hemichannel inhibitor carbenoxolone (Meigh et al., 2013) in P7–10 slices (100 μM Figures 1G–1I, *n* = 6). Carbenoxolone incubation did alter the electrophysiological properties of neurons (as previously reported in Tovar et al., 2009), but these changes would be expected to enhance the effects of hemi-channel opening rather than occlude them.

**Figure 2:**
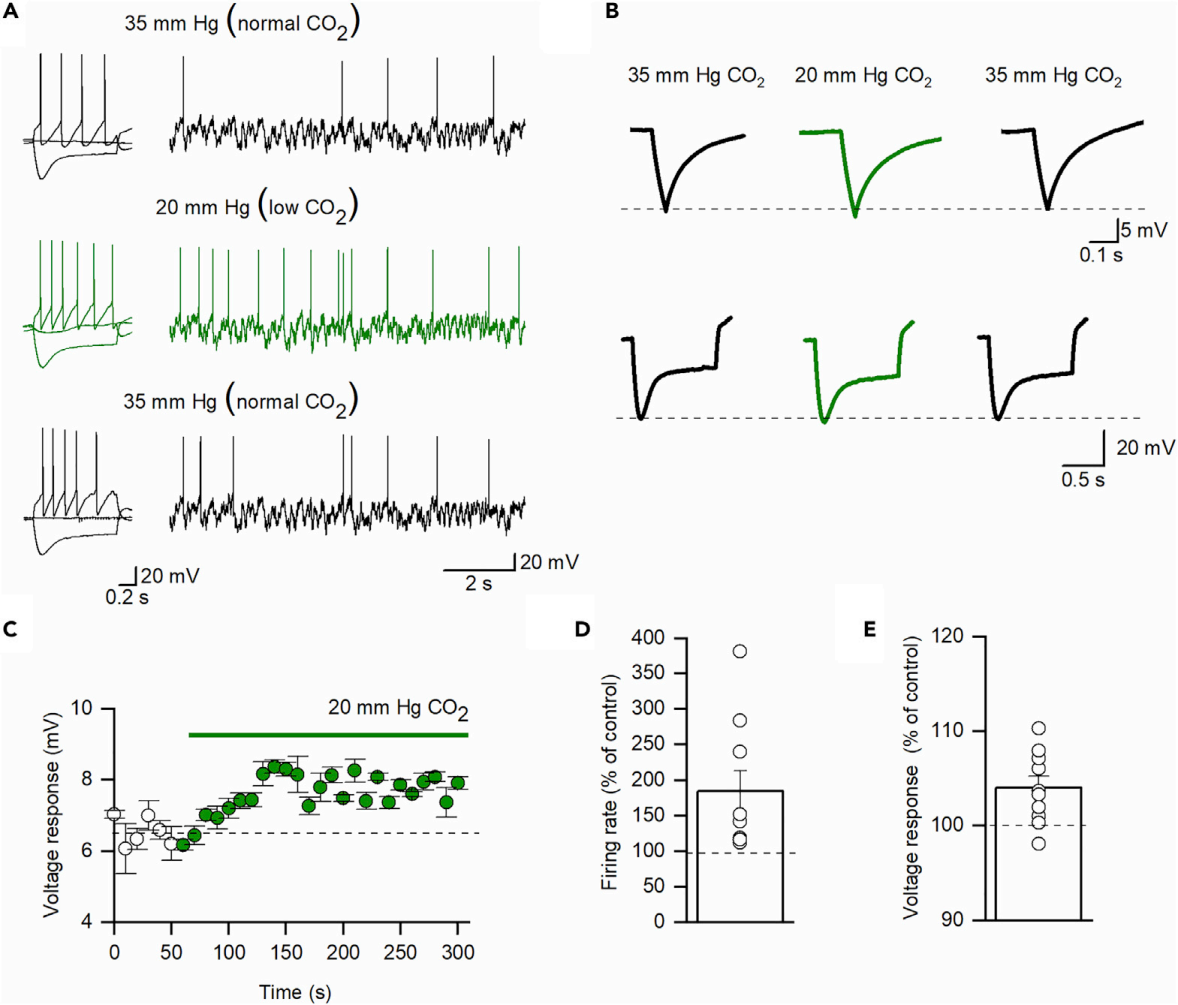
P7–10 Dopaminergic Neurons in the Substantia Nigra Are Sensitive to Lowering CO_2_ (A) An example recording from a P7 SN dopaminergic neuron. Black traces represent recordings in 35 mm Hg (normal CO_2_), and green traces represent recordings in 20 mm Hg (low CO_2_). An increase in firing rate can be observed (A) from the membrane potential responses to step and fluctuating current injections (as in Badel et al., 2008; see Methods). (B) The voltage response to a 50-pA hyperpolarizing step (top, traces are an average of 40 sweeps) and the membrane potential response to the −100-pA step injection (bottom). They show a small increase in voltage response due to increased input resistance with lowered CO_2._ Both the left and right panels show a partial recovery of both firing rate and input resistance after washing back into 35 mm Hg CO_2._ (C) Time course of changes in voltage response (P7–10, each point is a mean of six current steps, error bars are SEM) when CO_2_ was changed from 35 to 20 mm Hg. (D) Quantification of changes to firing rate as measured by the voltage response to fluctuating current input. (E) Quantification of the voltage response to hyperpolarizing step input. Both (D) and (E) display data normalized to the baseline values; raw data plots are available in Supplemental Information .

The midpoint for CO_2_-dependent opening of Cx26 hemichannels is around the basal level of PCO_2_ used in these experiments (35–40 mm Hg, Huckstepp et al., 2010a). Thus, a reduction in PCO_2_ should close Cx26 hemichannels leading to a decrease in resting conductance and a corresponding increase in firing rate. As predicted, in P7–10 SN DNs, decreasing PCO_2_ from 35 to 20 mm Hg (hypocapnia) increased the voltage response to hyperpolarizing current steps (104 ± 1.2% of control, p = 0.0078) and increased the firing rate (184 ± 28.65% of control, p = 0.015, Figure 2) consistent with a decrease in conductance. These effects of reduced CO_2_ were partially reversible (Figures 2A and 2B). Thus, small changes in CO_2,_ around normal resting levels (40 mm Hg), are sufficient to modulate SN DN excitability consistent with Cx26 hemichannel expression.

### Early Postnatal Substantia Nigra Dopamine Neurons Express Connexin 26 and Dye Load with Hypercapnia

**Figure 3:**
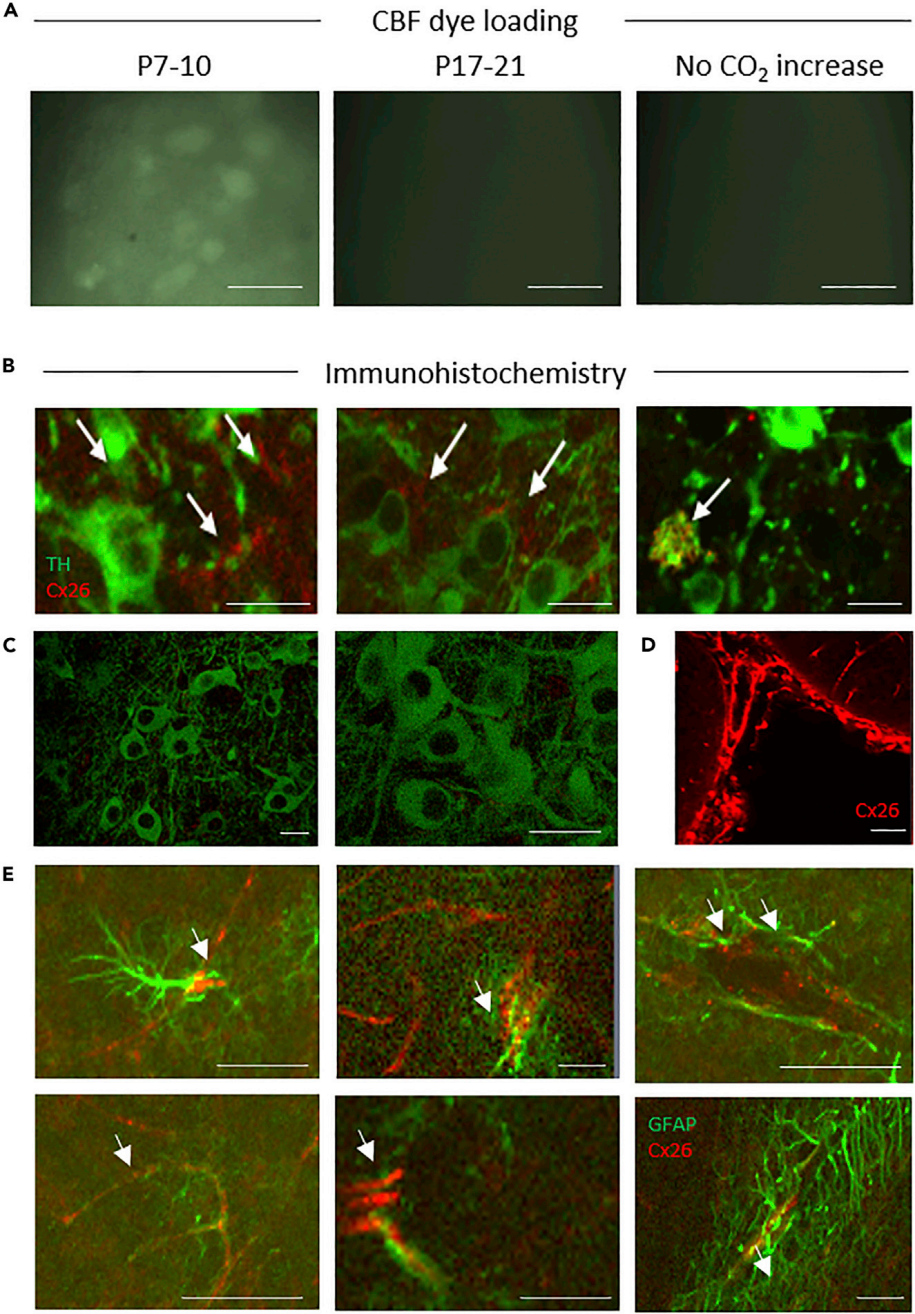
Dye Loading and Cx26 Expression in Substantia Nigra (A) Carboxyfluorescein (CBF) dye loading following hypercapnia in P7–10 slices (cell bodies are clearly labeled). No dye loading occurred if CO_2_ was not changed or in P17–21 slices; scale bar, 50 μM. (B) Immunofluorescent staining of P7–10 SN for Cx26 (red, arrows) in TH^+^ neurons (green). (C) No co-localization of Cx26 (red) in TH^+^ neurons (green) in the SN at P17–21; scale bars, 30 μM. Staining was deemed successful due to the positive leptomeninges staining from corresponding sections of the same brain. (D) Scale bar, 50 μM. (E) Cx26 (red, arrows) co-localized with GFAP (green, glial cell marker) in P17–21 SN; scale bar, 50 μM.

We next used a different, non-electrophysiological approach to provide further evidence that P7–10 SN DNs express CO_2_-sensitive hemichannels. A characteristic of hemichannels is that, when they open, they allow entry of membrane-impermeant fluorescent dyes into cells. Once the hemichannels close, the dye becomes trapped inside the cells (this is termed dye loading) and can then be used as a marker for cells that express CO_2_-sensitive hemichannels (Huckstepp et al., 2010a; Meigh et al., 2013). Grace and Bunney (1983) had previously shown that lucifer yellow can demonstrate dye coupling (into neighboring neurons from intracellular injection into a single neuron). However, Vandecasteele et al. (2006) attempted to dye load SN DNs (extracellular bath application, as described above) with lucifer yellow by opening hemichannels with low levels of Ca^2+^; this was unsuccessful. We decided to use the impermeant dye carboxyfluorescein (CBF) as we could be certain that it would pass through open Cx26 hemichannels as it has been shown in previous studies (Huckstepp et al., 2010a; Meigh et al., 2013) and confirmed that neurons in the SN of P7–10 mice (Figure 3A) could be loaded with the dye following hypercapnia. No dye loading occurred if the PCO_2_ was not increased. Dye loading did not occur in the SN of older mice (P17–21) or in CA1 hippocampal pyramidal cells (Figure S2). To further confirm that early postnatal SN DNs express connexin 26, we used a highly specific monoclonal antibody to Cx26 (Sun et al., 2009, Huckstepp et al., 2010a, 2010b). In slices from P7–10 mice, Cx26 expression was present in tyrosine hydroxylase-positive (TH^+^) neurons in the SN (Figure 3B). However, in older mice (P17–21), Cx26 appeared not to be expressed in TH^+^ neurons (Figure 3C). Cx26 was still expressed in the leptomeninges of corresponding sections P17–21 providing a positive control for the labeling protocol (Figure 3D). At P17–21, Cx26 sensitivity appeared to shift from TH^+^ cells to the neighboring glial cells (co-localizing with the glial marker GFAP, Figure 3E).

### Changes in CO_2_ Significantly Modifies the Excitability of Neurons in the VTA

**Figure 4:**
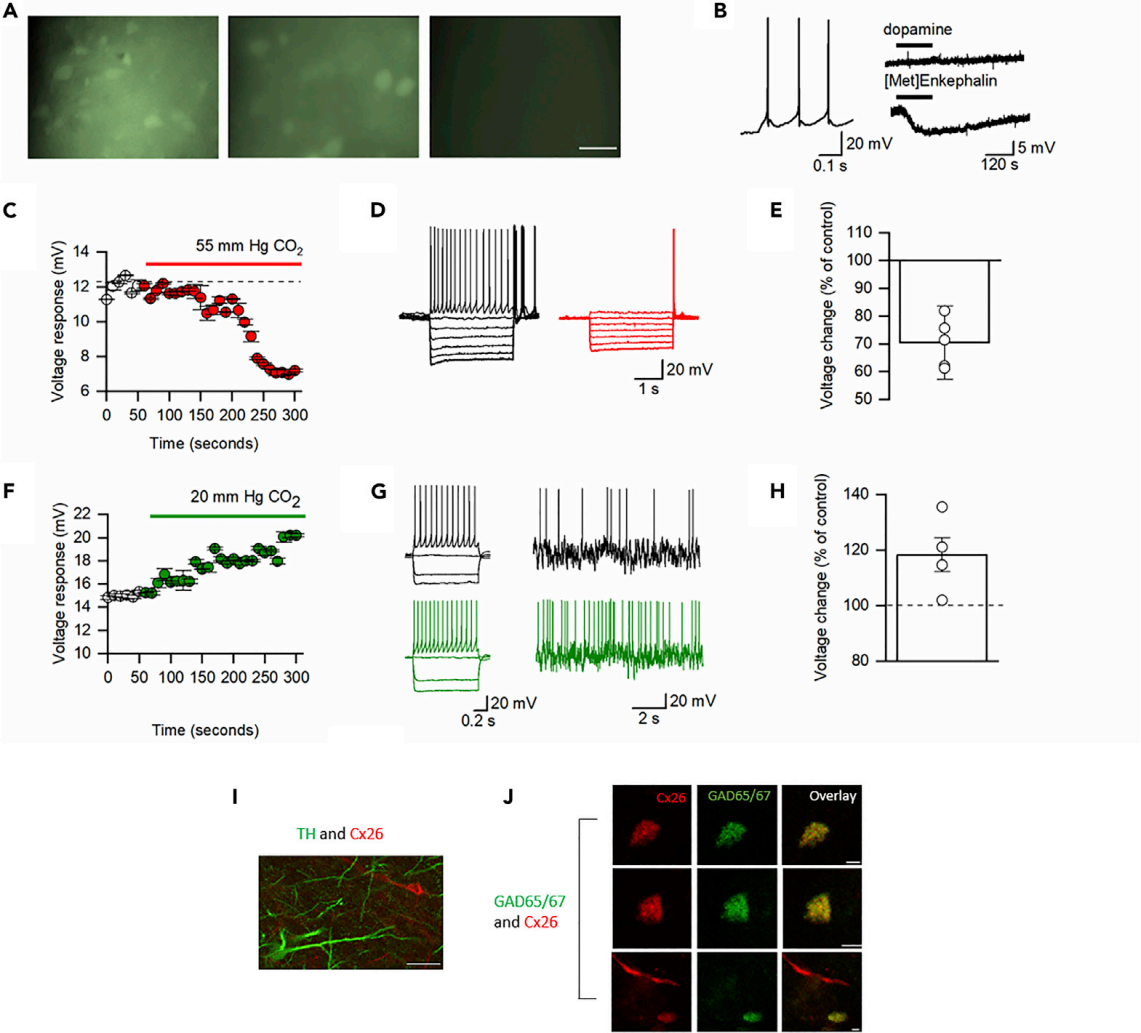
GABAergic Neurons in the VTA Are Sensitive to CO_2_ (A) CBF dye loading of VTA neurons in response to hypercapnia occurs at both P7–10 and P17–21 but does not occur without the increase in CO_2_ (hypercapnia), scale bar, 50 μM. (B) Characteristics of CO_2_-sensitive VTA neurons: firing pattern, hyperpolarization to the opioid receptor agonist [Met5]Enkephalin (10 μM) but not to dopamine (30 μM). (C) Time course of changes in voltage response (CO_2_ increased from 35 to 55 mm Hg, each point is a mean of six current steps, error bars are SEM). (D) Voltage responses to step currents at indicated time points in (C). (E) Quantification of changes in voltage response to increased CO_2_. (F) Time course of changes in voltage response (CO_2_ decreased from 35 to 20 mm Hg, each point is a mean of six current steps, error bars are SEM). (G) Voltage responses to step and fluctuating current inputs (as in Badel et al., 2008; see Methods) at indicated time points in (F) demonstrating increased input resistance and firing rate. (H) Quantification of changes in voltage response to decreased CO_2._ (I and J) Representative single optical planes immunohistochemistry images. (I) Immunofluorescent staining of P17–21 VTA for Cx26 (red), which is not expressed by TH^+^ neurons (green, no co-localization); scale bar, 50 μM. (J) Co-localization of Cx26 (red) with the soma of three individual GAD^+^ neurons (green) in the VTA (scale bar, 20 μM).

During the CBF loading assay carried out in the P17–21 slices, although no dye-filled neurons were observed in the SN, unexpectedly a population of dye-filled neurons was observed in the neighboring VTA (Figure 4A). This region is central to circuits controlling motivation, reward, and goal-directed behaviors (Morales and Margolis, 2017). Dye-loaded neurons in the VTA had a markedly different firing pattern and voltage response to current injection compared with SN DNs, were not hyperpolarized by dopamine, but were hyperpolarized by the opioid receptor agonist [Met5]Enkephalin, therefore, they could instead be GABAergic neurons (Johnson and North, 1992; Figure 4B). These VTA neurons showed electrophysiological changes similar to that observed for P7–10 SN DNs in response to changes in PCO_2_: increased PCO_2_ (55 mm Hg) decreased input resistance (voltage response reduced to 71 ± 13.2% of control p = 0.0055, 335 ± 66.7 to 222 ± 39.4 MΩ, p = 0.0446, before the sag *n* = 5) and firing rate (Figures 4C–4E). Reducing PCO_2_ to 20 mm Hg increased input resistance (voltage response increased to 118 ± 6.1%, p = 0.0428) and firing rate (193 ± 35%, p = 0.049, of control, Figures 4F–4H). To identify the phenotype of the CO_2_-sensitive neurons in the VTA, we carried out immunohistochemistry. Cx26 was not expressed in TH^+^ neurons in the VTA, so is not present in dopaminergic neurons (Figure 3I). However, Cx26 immunoreactivity was present in GAD65/67^+^ neurons (Figure 3J), a marker for GABAergic neurons in the VTA (Chieng et al., 2011), which fits with the electrophysiological properties of the CO_2_-sensitive neurons. Thus, the CO_2_-sensitive neurons in the VTA are GABAergic.

## Discussion

We have demonstrated an unexpected CO_2_-sensitive phenotype for neurons in the SN at P7–10 and in the VTA, with increases in CO_2_ markedly increasing their resting conductance. This effect appears to occur in only specific subtypes of neuron, as for example, it was not observed in hippocampal pyramidal cells. It is well established that increases in PCO_2_ can close gap junctions and that DN neurons in young animals are coupled (Connors et al., 1984; Bukauskas and Peracchia, 1997; Vandecasteele, 2005). However, this effect cannot account for the effects that we have observed. First, the increase in PCO_2_ that is required to close gap junctions is large and is well above the range of PCO_2_ changes we used to elicit effects on neuron electrophysiology. In addition, the closure of gap junctions would result in a decrease in whole-cell conductance and an increase in excitability, which is the opposite of what we observed in our study. Here we have provided several lines of evidence that suggest that our observations of an increase in CO_2_-sensitive conductance result from the opening of Cx26 hemichannels, whose open probability increases through the direct CO_2_-mediated carbamylation of lysine residues (Meigh et al., 2013). We have shown that the effects of CO_2_ in SN DNs occurs over the same developmental period as they express Cx26 mRNA (measured in an independent study, Vandecasteele et al., 2006). The effects of increasing PCO_2_ on resting conductance can be blocked by the hemichannel inhibitor carbenoxolone. Although carbenoxolone has neuronal and synaptic effects as well as blocking hemichannels, they would be expected to accentuate the observed increase in conductance rather than reducing it, therefore not obscuring the observations (Tovar et al., 2009). If the effects of PCO_2_ on cell conductance are due to the opening of Cx26 hemichannels, it would be predicted that, since the midpoint of Cx26 hemichannel opening lies around the basal level of PCO_2_ in our experiments (Meigh et al., 2013, 35 mM Hg), a decrease in CO_2_ would close Cx26 hemichannels leading to a decrease in the resting conductance. Such a decrease in conductance could be observed for both SN DN and VTA GABAergic neurons when CO_2_ was lowered.

SN dopaminergic neurons and VTA GABAergic neurons could be filled with a membrane-impermeant fluorescent dye (CBF) when PCO_2_ was increased (dye loading). CBF will pass through Cx26 hemichannels when they are open and then become trapped inside cells when the hemichannels are subsequently closed (Huckstepp et al., 2010a; Meigh et al., 2013). Unfortunately, CBF cannot be fixed with paraformaldehyde, which prevents the dye-filled neurons from being subsequently labeled using immunohistochemistry. However, we can be confident that the dye-filled cells were either SN DNs or VTA GABAergic neurons, as patch clamp recording was carried out before the dye loading (to confirm the identity of the cells from their electrophysiological properties and pharmacology) and then the same cells were subsequently dye filled. We have also used immunohistochemistry to show that Cx26 protein is expressed in these neurons. The expression pattern of Cx26 across development in SN DNs matched that reported for Cx26 mRNA expression (Vandecasteele et al., 2006). This particular Cx26 antibody (13-8100) has been used extensively to study the role of Cx26 in breathing. There are many independent papers that demonstrate the specificity of this antibody in KO of Cx26. KO of Cx26 in the organ of Corti abolishes Cx26 immunoreactivity with this antibody (Sun et al., 2009), and our prior publications show correspondence for Cx26 immunostaining with a reporter driven from the endogenous Cx26 promoter (Huckstepp et al., 2010a, 2010b).

To separate the effects of CO_2_ from any effects of changing pH, we kept extracellular pH constant during our experiments by using isohydric solutions (an increase in PCO_2_ under these conditions is termed isohydric hypercapnia). However, we did not measure intracellular pH and there will probably be transient changes in pH when the solutions are exchanged. It is well documented that intracellular pH will transiently acidify on application of the stimulus (raised PCO_2_) and transiently alkalinize on its removal (Filosa et al., 2002; Putnam, 2001). A mild intracellular acidification would be expected to result in hemichannel closure and therefore a decrease in conductance. Therefore, we concluded that our observations were not the result of a change in intracellular pH. In addition, these transient changes in pH cannot explain the marked and sustained changes in conductance that only occur in these specific subtypes of neuron.

In this paper we have outlined the CO_2_ sensitivity of specific neurons in the SN and VTA and provided a mechanism for this effect: Cx26 hemichannel expression. As far as we are aware this is the first documentation of neuronal expression of Cx26, which is usually found in glia (Nagy et al., 2011). Although we have carried out no behavioral analysis of the effects of CO_2_ sensitivity, it is interesting to speculate on its possible behavioral consequences. Since the mid-point of Cx26 opening lies around the resting level of CO_2_ in humans (Huckstepp et al., 2010a, 2010b; Meigh et al., 2013), small increases or decreases in CO_2_ will modulate neuron excitability and thus could potentially modulate behavior. This CO_2_ sensitivity switches from SN neurons to glia during early postnatal development but is retained in GABAergic neurons in the VTA. The switch from neuronal to glial expression could change the signaling direction from inhibitory to excitatory, as opening hemichannels in glia can allow the diffusion of molecules such as ATP, which could in turn excite SN DNs (through P2X or P2Y receptor activation). One speculated role for the CO_2_-mediated reduction in excitability of SN DNs in early postnatal life is, since the nest is likely to be hypercapnic, inhibition of movement may promote suckling behavior. The maintenance of CO_2_ sensitivity in the VTA postnatally is particularly interesting given its role in reward, addiction, motivation (Volkow and Morales, 2015), and sleep-wake behaviors (Eban-Rothschild et al., 2016). Activation of GABAergic neurons in the VTA induces sleep, and their inhibition increases wakefulness (Yu et al., 2018). There are several contributing mechanisms to hypercapnic arousal. The orexinergic neurons of the lateral hypothalamus, known to promote wakefulness, can be activated by hypercapnia, although this is through a pH-dependent transduction mechanism (Williams et al., 2007). The histaminergic neurons of the tuberomamillary nucleus (TMN), which also promote wakefulness, are activated by CO_2_ (Johnson et al., 2005; Anaclet et al., 2009). Neurons of the dorsal raphe, not involved in the control of breathing are pH/CO_2_ sensitive and contribute to hypercapnic arousal (Smith et al., 2018). Furthermore, the parabrachial nucleus integrates chemosensory inputs during hypercapnia from the medullary nuclei such as the retrotrapezoid nucleus and the raphe magnus, which contain pH-sensitive neurons to mediate arousal (Kaur et al., 2017). However, even after silencing these key relay neurons, hypercapnia still results in arousal albeit at a longer latency showing that other parallel pathways are involved (Kaur et al., 2017). Given that inhibition of the VTA GABAergic neurons have been demonstrated to cause wakefulness (Yu et al., 2018) we hypothesize that inhibition of these neurons by modestly raised CO_2_ could potentially contribute an additional parallel pathway of hypercapnic arousal.

### Limitations of the Study

This report outlines the novel observation of CO_2_ sensitivity in a specific subset of neurons with several lines of evidence that it results from Cx26 hemichannel expression. There is no data on the physiological significance of this CO_2_ sensitivity, in particular regarding movement and reward behavior. This will be examined in future studies. There is no quantification of the expression pattern of Cx26 protein in either TH^+^ or GAD^+^ neurons. In the future, tools like fluorescence in situ hybridization (FISH) could be used to produce more accurate measurements of expression.

### Resource Availability

#### Lead Contact

Further information and requests for resources and reagents should be directed to and will be fulfilled by Mark Wall (Mark.Wall@warwick.ac.uk) or Emily Hill (E.hill.2@warwick.ac.uk).

#### Materials Availability

This study did not generate new unique reagents.

#### Data and Code Availability

This study did not generate new code or structural datasets.

## Methods

All methods can be found in the accompanying Transparent Methods supplemental file.

## Supplemental Information

Document S1. Transparent Methods, Figures S1–S8, and Tables S1–S5

## Footnotes

Supplemental Information can be found online at https://doi.org/10.1016/j.isci.2020.101343.
