# Supplementary material for: Moderate Changes in CO2 Modulate the Firing of Neurons in the VTA and Substantia Nigra
Source: iScience. 2020 Jul 4;23(7):101343. doi: 10.1016/j.isci.2020.101343 (PMC7371905; doi:10.1016/j.isci.2020.101343)
Supplement: Document S1. Transparent Methods, Figures S1–S8, and Tables S1–S5 [file mmc1.pdf]

iScience, Volume *23*

Supplemental Information

Moderate Changes in CO_2 Modulate the Firing of Neurons in the VTA and Substantia Nigra

Emily Hill, Nicholas Dale, and Mark J. Wall

Supplementary Data

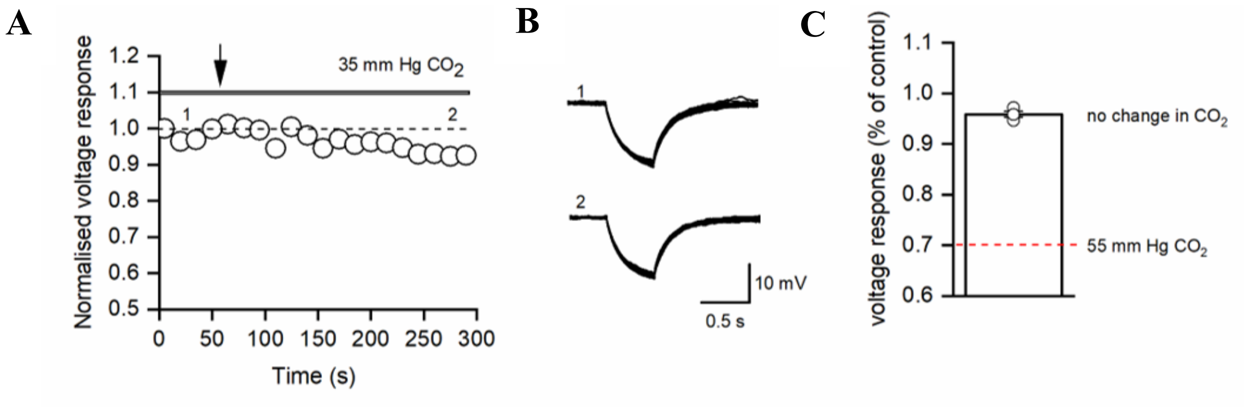

Figure S1. Control for stability of recordings and for changing solution but maintaining constant PCO_2, related to figure 1. (A) Graph plotting the voltage response to 50 pA hyperpolarising current steps from a P10 SN DN with each point the mean of 15 sweeps. The CO_2 was maintained at 35 mm Hg but solutions were exchanged (arrow) to ensure that the observations are not an artefact of the solution switching process. This also illustrates the stability of voltage responses throughout the recording. (B) Associated voltage traces (50 superimposed traces) in response to step currents at the indicated time points from A. (C) Quantification of the voltage response changes when the solution was exchanged, relative to the amplitude of the response at whole cell breakthrough, data is presented as mean ± SEM (points are from individual experiments). Red dashed line represents the decrease in response for cells which were exposed to high CO_2 (55 mm Hg) after an equal amount of time. There is no effect of changing solution and the voltage response is stable over the duration of the recording.

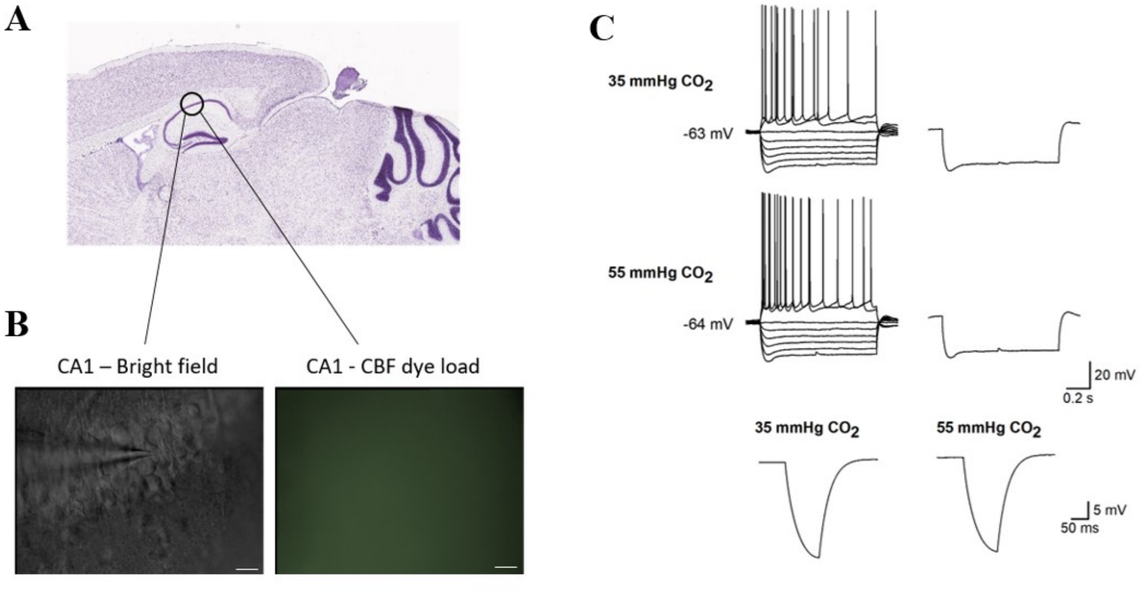

Figure S2: CA1 pyramidal cells show no dye loading or electrophysiological changes in response to high CO_2, related to figure 1 (A) Localisation of CA1 region of the hippocampus in a sagittal slice (Adapted from Allen Mouse Brain Atlas, 2004). (B), (left) Bright-field image of the CA1 region demonstrating the location of a recorded pyramidal cell (scale bar = 30 µM). The slice was then subjected to carboxy-fluorescein (CBF) dye-loading (see methods) as used for the SN and VTA. There was no visible dye loading of the neurons (right). (C), (Top) Membrane potential traces recorded from a CA1 pyramidal neuron in response to current steps (3 s steps starting at -200 pA, increasing by 50 pA until there is a regular firing pattern) in 35 mmHg CO_2. (Inset) Single membrane potential trace in response to the injection of -200 pA (3 s) in 35 mmHg CO_2. (Bottom) Membrane potential traces recorded from the same CA1 pyramidal neuron in response to current steps in 55 mmHg CO_2. Single membrane potential trace in response to a -200 pA (3 s) current step in 55 mmHg CO_2.

Table S1: Comparison of the electrophysiological parameters of P7-10 and P17-21 SN dopaminergic neurons, related to figure 1.

	Rin (pre-sag) MΩ			Rin Steady MΩ			RMP		
	**Mean**	**SEM**	**95% CI**	**Mean**	**SEM**	**95% CI**	**Mean**	**SEM**	**95% CI**
**P7-10**	380.4	± 28.16	± 55.2	246.9	± 24.63	± 48.3	-59.3	± 1.33	± 2.6
**P17-21**	289.8	± 22.45	± 44	193.9	± 42.85	± 84	-60	± 2.08	± 4.07

Rin Steady: Mann-Whitney non parametric test p = 0.3037 ns

Rin Pre Sag: Mann-Whitney non parametric test: p = 0.0977 ns

RMP: Mann- Whitney non parametric test p = 0.8352 ns

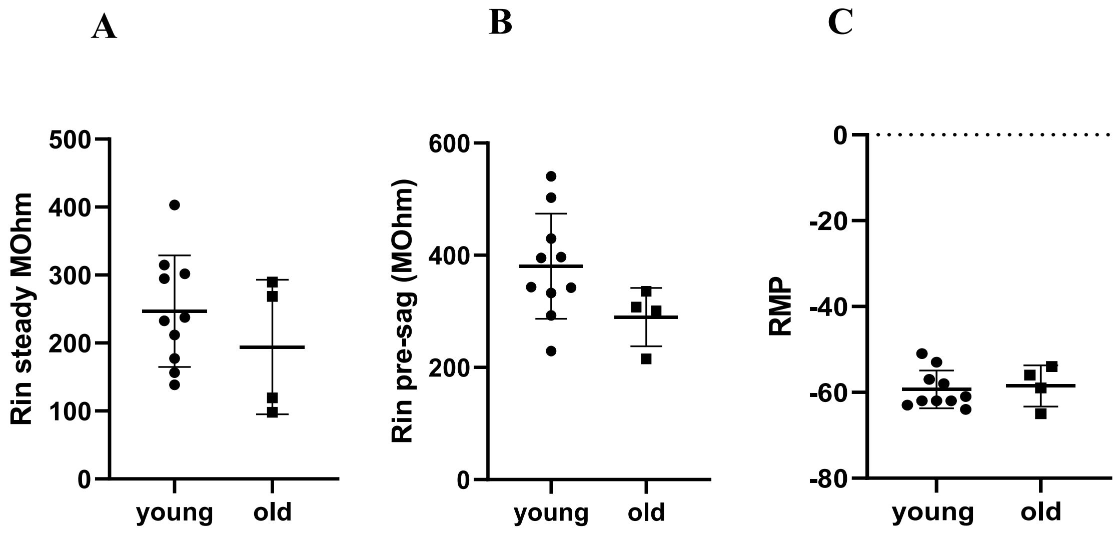

Figure S3: Comparison of the electrophysiological parameters of P7-10 and P17-21 SN neurons, related to figure 1.

Input resistance measurements 'both before the sag' (A) and at 'steady state' (B) decreased during development in line with published studies (4). We saw no difference in the stability of recordings and there was no change to resting membrane potential (C).

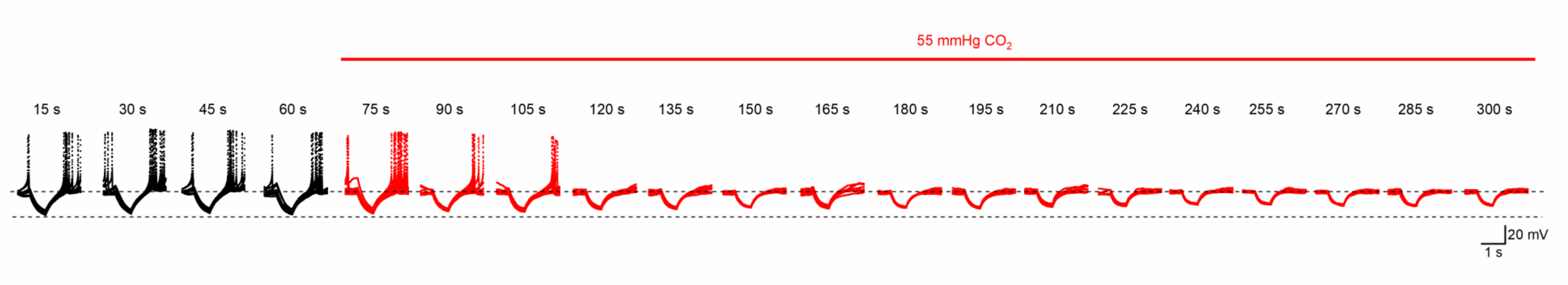

Figure S4: Example raw data traces for the full timeframe of raised CO_2 exposure, related to figure 1. A representative example from a P7-10 substantia nigra dopaminergic neuron. Membrane potential traces in response to -50 pA current steps in 35 mmHg CO_2 (black), then switched over to 55 mmHg CO_2 (red). Each plot represents a timepoint from the graph in Figure 1B and displays 9 overlaid sweeps within each 15 second time interval. A clear reduction in voltage response can be observed over time, as is summarised in Figure 1B-C.

Raw data plots

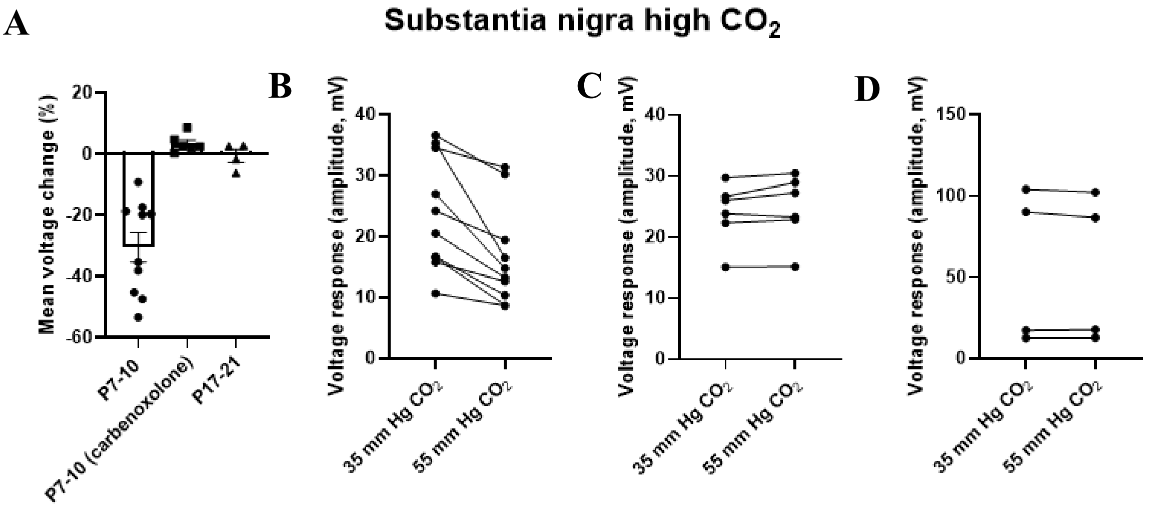

Figure S5: Raw data plots for raising CO_2 in the substantia nigra, related to figure 1 Quantification of voltage response changes (35 to 55 mm Hg CO_2), replicated from 1I. B-D represent the amplitude of the voltage response to a 50-pA hyperpolarising step current injection, in 35 mm Hg CO_2 and 55 mm Hg CO_2. Data points from each experiment is joined up by a line to represent that they are paired. It can be clearly observed in (B) that there is a decrease in the amplitude of the voltage response for P7-10 mice, which is not replicated in the presence of carbenoxolone (C) or in older mice (P17-21, D).

Table S2: Statistical analysis of data on raising CO_2 in the substantia nigra, related to figure 1

SN P7-10, 35 mm Hg **vs** 55 mm Hg	p=0.0020
SN P7-10 (carbenoxolone), 35 mm Hg **vs** 55 mm Hg	p=0.0938
SN P17-21, 35 mm Hg **vs** 55 mm Hg	p=0.3302
**Kruskal-Wallis ANOVA:**	p<0.0001
SN P7-10 **vs** P7-10 (carbenoxolone) **vs** P17-21	
**Dunn's multiple comparisons:**	p=0.0014
P7-10 **vs** P7-10 (carbenoxolone)	p=0.0304
P7-10 **vs** P17-21	p>0.9999
P7-10 (carbenoxolone) **vs** P17-21	

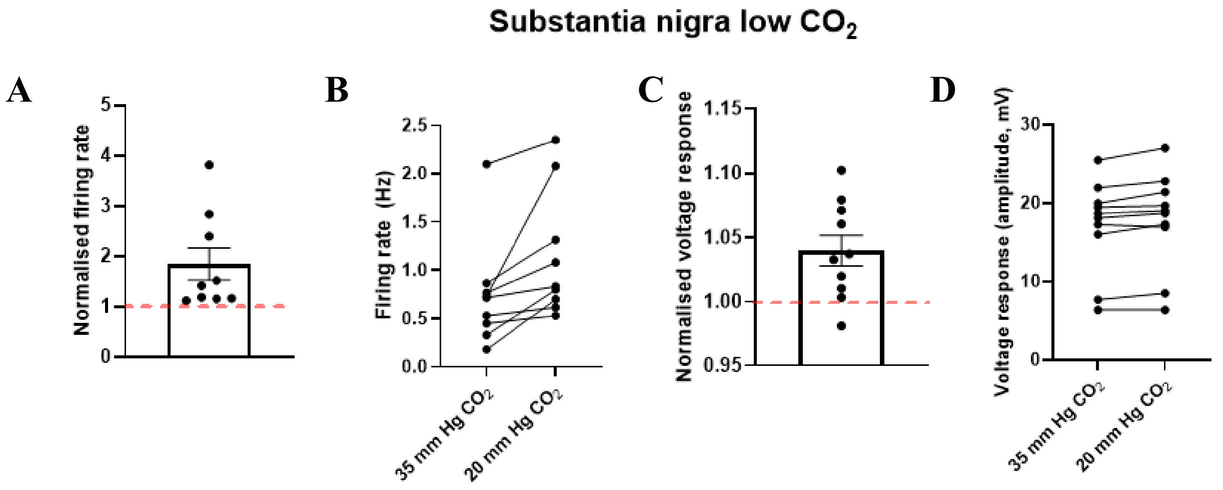

Figure 6: Raw data plots for lowering CO_2 in the substantia nigra, related to figure 2. A. Quantification of the firing rate changes (35 mm Hg to 20 mm Hg CO_2), replicated from 2D. B. Raw firing rate data, in 35 mm Hg CO_2 and 20 mm Hg CO_2. C. Quantification of voltage response changes (35 to 20 mm Hg CO_2), replicated from 2E. D. The amplitude of the voltage response to a 50-pA hyperpolarising step current injection, in 35 mm Hg CO_2 and 20 mm Hg CO_2. In B and D, data points from each experiment is joined up by a line to represent that they are paired. An increase in firing rate and voltage response are observed.

Table S3: Statistical analysis of data on lowering CO_2 in the substantia nigra, related to figure 2

SN Firing rate, 35 mm Hg **vs** 20 mm Hg	$p=0.0039$
SN Voltage response 35 mm Hg **vs** 20 mm Hg	$p=0.0098$

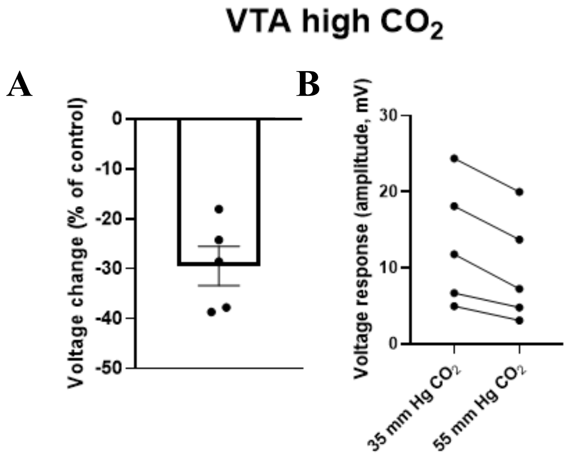

Figure S7: Raw data plots for raising CO_2 in the ventral tegmental area, related to figure 4. A. Quantification of voltage response changes (35 to 55 mm Hg CO_2), replicated from 3E. B. The amplitude of the voltage response to a 50-pA hyperpolarising step current injection, in 35 mm Hg CO_2 and 55 mm Hg CO_2. Data points from each experiment is joined up by a line to represent that they are paired. A decrease in voltage response can be observed.

Table S4: Statistical analysis of the data on raising CO_2 in the ventral tegmental area, related to figure 4

VTA Voltage response 35 mm Hg **vs** 55 mm Hg	p=0.0055

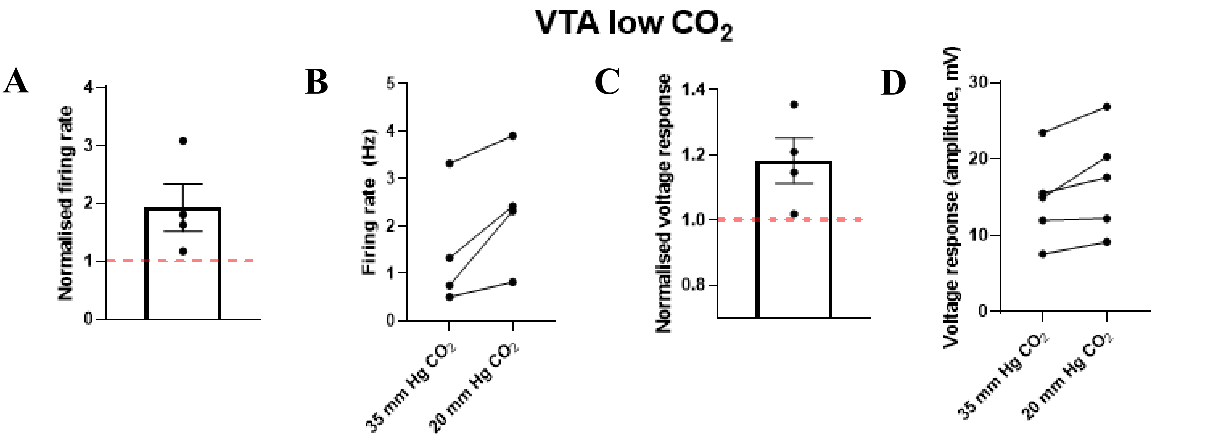

Figure S8: Raw data plots for lowering CO_2 in the ventral tegmental area, related to figure 4 A. Quantification of the firing rate changes (35 mm Hg to 20 mm Hg CO_2). B. Raw firing rate data, in 35 mm Hg CO_2 and 20 mm Hg CO_2. C. Quantification of voltage response changes (35 to 20 mm Hg CO_2), replicated from 3H. D. The amplitude of the voltage response to a 50-pA hyperpolarising step current injection, in 35 mm Hg CO_2 and 20 mm Hg CO_2. In B and D, data points from each experiment is joined up by a line to represent that they are paired. An increase in firing rate and voltage response are observed. Increases to both firing rate and voltage response can be observed.

Table S5: Statistical analysis of the data on lowering CO_2 in the ventral tegmental area, related to figure 4

VTA Voltage response 35 mm Hg **vs** 55 mm Hg	p=0.0428
VTA Firing Rate 35 mm Hg **vs** 55 mm Hg	p=0.0490

Transparent methods

KEY RESOURCES TABLE

REAGENT or RESOURCE	SOURCE	IDENTIFIER
**Antibodies**		
Sheep polyclonal Anti-Tyrosine hydroxylase	Merck	AB1542
Mouse monoclonal anti-Connexin 26	Invitrogen	138100
Chicken polyclonal anti-GFAP	Abcam	ab4674
Rabbit polyclonal anti-GAD65 + GAD67	Abcam	ab49832
Donkey anti-sheep 594	Invitrogen	A11016
Donkey anti-mouse 594	Invitrogen	A21203
Donkey anti-sheep 488	Invitrogen	A11015
Goat anti-chicken 488	Invitrogen	A11039
Goat anti-Rabbit 488	Invitrogen	A11008
**Chemicals, Peptides, and Recombinant Proteins**		
Met5[enkephalin]	Merck	M6638
Carbenoxolone disodium salt	Sigma Aldrich	C4790-1G
Dopamine Hydrochloride	Sigma Aldrich	H8502-5G
(6)-Carboxy-fluorescein (CBF)	Novabiochem	8.51082.001
Alexa Fluor 594 hydrazide	Molecular Probes	10072752
**Software and Algorithms**		
pClamp	http://www.moleculardevices.com/products/software/pclamp.html	RRID:SCR_011323
Zen Black	http://www.zeiss.com/microscopy/en_us/products/microscope-software/zen.html#introduction	RRID:SCR_013672
Origin	http://www.originlab.com/index.aspx?go=PRODUCTS/Origin	RRID:SCR_014212

Methods

Preparation of acute brain slices

All experiments were approved by the local Animals Welfare and Ethics Board (AWERB) at the University of Warwick. C57/Bl6 mice from two age groups (P7-10 and P17-21) were killed by cervical dislocation and decapitated in accordance with the U.K. Animals (Scientific Procedures) Act (1986). The brain was rapidly dissected and kept on ice. The cerebellum was removed, and the rostral section of the brain was trimmed. The brain was then mounted rostral side down. Coronal slices (350 μM) were cut with a Microm HM 650V microslicer in cold (2-4°C) high Mg^{2+}, low Ca^{2+} aCSF, composed of (mM): 127 NaCl, 1.9 KCl, 8 $MgCl_2$, 0.5 $CaCl_2$, 1.2 KH_2PO_4, 26 $NaHCO_3$, 10 D-glucose (pH 7.4 when bubbled with 95% O_2 and 5% CO_2, 300 mOSM). Slices were stored at 34 °C in standard aCSF (1 mM Mg^{2+} and 2 mM Ca^{2+}) for 1 to 8 hours.

Whole-cell patch clamp recording

A slice was transferred to the recording chamber, submerged and perfused (2-3 ml/min^{-1}) with aCSF at 30 °C. Slices were visualized using IR-DIC optics with an Olympus BX151W microscope (Scientifica, Bedford UK) and a CCD camera (Hitachi). Whole-cell current-clamp recordings were made from neurons in the substantia nigra, ventral tegmental area or from CA1 pyramidal neurons in the hippocampus using patch pipettes (5–10 MΩ) manufactured from thick walled glass (Harvard Apparatus, Edenbridge, UK). Intracellular solution was filtered before use (0.2 μm) and contained in (mM): potassium gluconate 135, NaCl 7, HEPES 10, EGTA 0.5, phosphocreatine 10, MgATP 2, NaGTP 0.3 293 mOSM, pH 7.2). A subset of neurons were filled with AF594 dye (50 μM) via the patch pipette for immunohistochemistry. Voltage recordings were made using an Axon Multiclamp 700B amplifier

(Molecular Devices, USA) and digitised at 20 KHz. Data acquisition and analysis were performed using pClamp 10 (Molecular Devices). Recordings from neurons that had a resting membrane potential of between -55 and – 75 mV at whole-cell breakthrough were accepted for analysis. The bridge balance was monitored throughout the experiments and any recordings where it changed by more than 20 % were discarded.

Solutions are based on Huckstepp et al (2010)

Control (35 mmHg CO_2) aCSF contained in (mM): NaCl 124, $NaHCO_3$ 26, NaH_2PO_4 1.25, KCl 3, D-glucose 10, $MgSO_4$ 1, $CaCl_2$ 2, bubbled with 95%O_2 5% CO_2 with a final pH of ~7.4.

Hypercapnic (55 mmHg CO_2) aCSF contained in (mM): NaCl 100, $NaHCO_3$ 50, NaH_2PO_4 1.25, KCl 3, D-glucose 10, $MgSO_4$ 1, and $CaCl_2$ 2. Solution was saturated with 9% CO_2 (with the balance being O_2) with pH maintained to match control (35 mm Hg).

Hypocapnic (20 mm Hg CO_2) aCSF contained in (mM): NaCl 140, $NaHCO_3$ 10, NaH_2PO_4 1.25, KCl 3, D-glucose 10, $MgSO_4$ 1 and $CaCl_2$ 2. Solution was saturated with 2% CO_2 (with the balance being O2), with pH maintained to match control (35 mm Hg).

Stimulation Protocols

Standard IV protocol

A standard current-voltage relationship was constructed by injecting step currents (3 s duration, every 5 s) starting at -200 pA and then incrementing by either 50 or 100 pA until a regular firing pattern was induced. A plot of step current against voltage response around the resting potential was used to measure the input resistance (gradient of the fitted line).

Naturalistic current injection

The naturalistic current was generated using the summed numerical output of two Ornstein–Uhlenbeck processes (Uhlenbeck & Ornstein, 1930) with time constants τfast = 3 ms and τslow = 10 ms. This naturalistic current waveform (as in Badel et al, 2008), which mimics the stochastic actions of AMPA and GABA-receptor channel activation, was injected into cells (40 s duration) and the resulting voltage recorded (as a fluctuating noisy trace). This voltage trace was then used to evaluate the frequency of action potential firing.

Current Injection to assess conductance changes

A hyperpolarising step of 50 pA (100 ms) was injected at a frequency of 1 Hz. This allowed the time course of changes in input resistance/conductance to be assessed. For analysis, averages were constructed for 10-minute periods in 35 mm Hg and 55 mmHg CO_2 (when the effects of CO_2 had reached steady state).

For each recording, once whole cell breakthrough had occurred cells were allowed to equilibrate for a few minutes. Following this a standard IV curve was constructed and naturalistic current traces were injected to enable the measurement of firing rate. After these measurements had been recorded (5-10 mins post- whole cell breakthrough), the hyperpolarising step current was initiated to look for voltage changes in response to altered levels of carbon dioxide (from 35 mm Hg to 55 mm Hg, isohydric).

Immunohistochemistry

Mice (P7-10 and P17-20) were cardiac perfused with 4% PFA and then post-fixed overnight at 4ºC. The tissue was washed with PBS and then sliced coronally (350 μm). The slices were left to recover for 1 hour and then were blocked for an hour (1% BSA, 0.4% Triton 100X in PBS, 400 μl per slice) then washed 3 times for 5 minutes with PBS. The primary antibodies against tyrosine hydroxylase, (1:1000, Sheep), GFAP (1:1000, Chicken) or GAD65/67 (1:1000, Rabbit) and the primary antibody against connexin 26 (1:200, Mouse) were added to the slices (400 μl per slice) for an hour at room temperature and then kept at 4-8ºC overnight. Slices were washed 5 times for 5 minutes with PBS and the corresponding secondary antibody (anti-sheep 488, 1:500, anti-mouse 594, anti-sheep 594, anti-chicken 488 or anti-rabbit 488, 1:500, 400 μl per slice) added for 4 hours at room temperature. The slices were then washed 5 times for 5 minutes with PBS, and then mounted on glass slides with Vectashield (Vector laboratories, Peterborough UK). All imaging was carried with confocal microscopy (Leica 710 and Zen Black for image acquisition and processing). Controls were carried out without the primary antibodies and showed no fluorescence.

Dye loading

The dye loading method is based on that described in Huckstepp et al (2010). Briefly, a slice was transferred to the recording chamber, submerged and perfused (2-3 ml/min^{-1}) with control aCSF (35 mmHg CO_2) at 30 °C. Slices were visualized using IR-DIC optics with an Olympus BX151W microscope (Scientifica, Bedford UK) and a CCD camera (Hitachi). To confirm the correct location for imaging, whole cell patch clamp recordings were used to identity DNs and GABAergic neurons in the SN and VTA (Fig. 1 and 4). Slices were then allowed to equilibrate for 20 minutes. The control aCSF was then exchanged for 55 mmHg CO_2 aCSF (hypercapnic) containing 5(6)-carboxy-fluorescein (CBF, 100 μM) for 20 mins to allow the CO_2 sensitive-hemichannels to open. The solution was then exchanged for 35 mmHg CO_2 aCSF containing CBF (100 μM) for 5 minutes to allow the hemichannels to close. Finally, the slice was washed with 35 mmHg CO_2 aCSF for 3 hours to reduce the background staining before imaging. Images were taken using the CCD camera (Hitachi) with 488 nm fluorescence (CoolLED). As CBF rapidly bleaches, images were quickly acquired from regions of interest. CBF cannot be fixed using PFA (as it lacks the required groups for cross-linking).

Statistics

Data is represented as mean and standard error of the mean with individual experiments represented by single data points. Appropriate statistical tests were chosen based on sample size, whether there were repeated measures and whether the populations were paired or unpaired (Wilcoxon rank sum/ paired t-tests and Mann Whitney tests respectively). For tests of more than two variables, Kruskal-Wallis ANOVAs were run with Dunn's post hoc multiple comparisons. All tests were run to find significance at the level $p < 0.05$ and were performed on raw (non-normalised) data, available in supplementary data (figures 5-8).
